# Cadherin‐6 is a novel mediator for the migration of mesenchymal stem cells to glioblastoma cells in response to stromal cell‐derived factor‐1

**DOI:** 10.1002/2211-5463.13815

**Published:** 2024-05-08

**Authors:** Aran Park, Seung‐Eun Kim, Jinyeong Yu, Donghyun Son, Kyung‐Sup Kim, Eunjin Koh, Ki‐Sook Park

**Affiliations:** ^1^ Graduate School of Biotechnology Kyung Hee University Yongin Korea; ^2^ Department of Biomedical Science and Technology, Graduate School Kyung Hee University Seoul Korea; ^3^ Industry‐Academic Cooperation Foundation Kyung Hee University Seoul Korea; ^4^ Department of Biochemistry and Molecular Biology, College of Medicine Yonsei University Seoul Korea; ^5^ East‐West Medical Research Institute Kyung Hee University Seoul Korea

**Keywords:** bone marrow‐derived mesenchymal stem cells, cadherin‐6, glioblastoma, migration, stromal cell‐derived factor‐1

## Abstract

Glioblastoma recruits various nontransformed cells from distant tissues. Although bone marrow‐derived mesenchymal stem cells (MSCs) have been observed migrating to glioblastoma, the underlying mechanism driving MSC migration toward glioblastoma remains unclear. Tumor vascularity is critical in the context of recurrent glioblastoma and is closely linked to the expression of stromal cell‐derived factor‐1 (SDF‐1). We demonstrated that cadherin‐6 mediated MSC migration both toward SDF‐1 and toward glioblastoma cells. Cadherin‐6 knockdown resulted in the downregulation of MSCs capacity to migrate in response to SDF‐1. Furthermore, MSCs with cadherin‐6 knockdown exhibited impaired migration in response to conditioned media derived from glioblastoma cell lines (U87 and U373) expressing SDF‐1, thus simulating the glioblastoma microenvironment. Moreover, MSCs enhanced the vasculogenic capacity of U87 cells without increasing the proliferation, cancer stem cell characteristics, or migration of U87. These results suggest that the current strategy of utilizing MSCs as carriers for antiglioblastoma drugs requires careful examination. Furthermore, cadherin‐6 may represent a novel potential target for controlling the recruitment of MSCs toward glioblastoma.

## Graphical abstract

Cadherin‐6 directs bone marrow‐derived mesenchymal stem cells (MSCs) to glioblastoma cells in response to SDF‐1. Knockdown of Cadherin‐6 hampers MSC migration toward the glioblastoma microenvironment. MSCs boost the vasculogenic capacity of glioblastoma cells, like U87, prompting careful consideration in therapy. Cadherin‐6 stands out as a promising target for regulating MSC recruitment to glioblastoma.

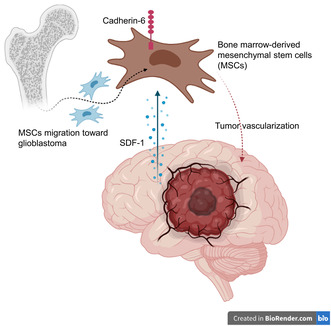

**Abbreviations**
3D cell migration: three‐dimensional cell migration
CON CM: the control conditioned media
DF: human dermal fibroblasts
MSCs: bone marrow‐derived mesenchymal stem cells
U373 CM: the conditioned media derived from U373 cells
U87 CM: the conditioned media derived from U87 cells
U87: U‐87 MG

Glioblastoma is the most lethal type of malignant brain tumor. In recent years, the tumor microenvironment has been recognized as playing an important role in glioblastoma and as a therapeutic target [1]. Tumor cells interact with various nontumor cells, including those exhibiting characteristics resembling mesenchymal stem cells [1]. Some of the mesenchymal stem cell‐like cells are mesenchymal stem cells recruited from the bone marrow [2]. Mesenchymal stem cells are known to be recruited from the bone marrow to distant tissues that express stromal cell‐derived factor‐1 (SDF‐1) [3]. Radiation delivered in the treatment of glioblastoma upregulated the expression of SDF‐1 in the glioblastoma, and SDF‐1 then led to the recruitment of bone marrow cells such as myelomonocyte [4]. SDF‐1‐dependent recruitment of bone marrow cells was associated with the increase in the functional tumor vascularity [4, 5]. The mesenchymal stem cell‐like cells of glioblastoma microenvironment regulate tumor cell proliferation, maintenance of cancer stem cells, and angiogenesis [2, 6]. Moreover, there is an inverse association between the frequency of mesenchymal stem cell‐like cells in glioblastoma and patient survival [2, 7]. Hence, it is crucial to identify the intrinsic molecular mechanisms responsible for the recruitment of bone marrow‐derived mesenchymal stem cells (MSCs) into glioblastoma and their subsequent migration into neighboring cells when developing therapeutic strategies for glioblastoma. However, underlying intrinsic molecular mechanisms for these processes remain undiscovered.

The cadherin superfamily orchestrates various biological processes, including migration, proliferation, and morphogenesis, as well as cell–cell adhesion for normal epithelial homeostasis [8, 9]. Cadherins also play a crucial role in pathological conditions, such as tumor progression. The dysregulation of cadherins is associated with major oncogenic pathways, which induce epithelial–mesenchymal transition (EMT) and uncontrolled proliferation [10, 11]. Furthermore, cadherins contribute to forming tumor microenvironment. The loss of p120 catenin, which interacts with the juxtamembrane domain of E‐cadherin, leads to an increase in immune cell infiltration, resulting in the formation of tumor microenvironment [12]. Tumor cells recruit MSCs, which can then differentiate into various cell types within the tumor microenvironment, supporting tumor cells through mechanisms like promoting tumor vascularity. N‐cadherin plays a role in mediating the collective migration of mesenchymal stem cells toward both breast tumor cells and prostate tumor cells [13, 14].

Cadherin‐6 is a member of the cadherin superfamily. Cadherin‐6 has been known to participate in the morphogenesis of neural tissue and kidney during embryonic development [15, 16]. The oncogenic roles of cadherin‐6 have been also identified [17, 18]. Cadherin‐6 expression increases in gastric tumor tissue compared with normal gastric tissue, and the aberrant expression of cadherin‐6 is associated with poor clinical outcomes in gastric cancers [17]. However, the role of cadherin‐6 in forming the tumor microenvironment has been remained unidentified.

In the current study, we have newly identified cadherin‐6 as an intrinsic molecule responsible for mediating the migration of MSCs toward glioblastoma cells. Additionally, our research has demonstrated that MSCs enhance the vasculogenic characteristics of glioblastoma cells by increasing the expression of angiogenic factors, such as vascular endothelial growth factor (VEGF).

## Materials and methods

### Cell culture

Human bone marrow‐derived mesenchymal stem cells (MSCs), human umbilical vein endothelial cells (HUVECs), and human dermal fibroblasts (DF) were acquired from Lonza (Basel, Switzerland). HEK293T cells and MDA‐MB‐231 cells were obtained from the American Type Culture Collection (ATCC, Manassas, VA, USA). U373 and HEK293 cells were obtained from the Korean Cell Line Bank (KCLB, Seoul, Korea). U‐87 MG (U87) cells were obtained both from ATCC and KCLB. To indirectly co‐culture U87 and MSCs, culture inserts containing MSCs were placed onto U87 and incubated for 3 days.

### Conditioned media preparation

Conditioned media were prepared by incubating the cells under the serum‐free conditions for 3 days. To prepare conditioned media for the control group, the media from each cell type were incubated for the same duration under cell‐free conditions.

### Transfection of cells with small interfering RNAs (siRNA)

Lipofectamine RNAiMAX (Thermo Fisher Scientific, Waltham, MA, USA) was used for siRNA transfection into MSCs according to the manufacturer's instructions. Trilencer‐27 Universal scrambled negative control siRNA (control siRNA, ORIGENE, Rockville, MD, USA) was used as a negative control. siRNA of cadherin‐6 was synthesized by Genolution (Seoul, Korea).

### Reverse transcription polymerase chain reaction (RT‐PCR) and real‐time quantitative reverse transcription PCR (RT‐qPCR)

Total RNA extraction was carried out utilizing TRIzol reagent (Thermo Fisher Scientific), followed by cDNA synthesis using SuperScript III Reverse Transcriptase (Thermo Fisher Scientific), following the manufacturer's protocols. Conventional PCR was performed using IP‐Taq PCR Mastermix (COSMOGENTECH, Seoul, Korea). RT‐qPCR was conducted using the SYBR Green reagent (Thermo Fisher Scientific). The human ribosomal protein S9 gene (RPS9) was utilized as an endogenous reference. The sequences of primers are available in Table 1.

**Table 1:** Sequences of primers.

	Gene name	Sense	Antisense
Conventional PCR			
Ribosomal protein S9	RPS9	5′‐CTGACGCTTGATGAGAAGGAC‐3′	5′‐CAGCTTCATCTTGCCCTCAT‐3′
SDF‐1	CXCL12	5′‐CGCCATGAACGCCAAGGTCGTGGTCG‐3′	5′‐GGCTGTTGTGCTTACTTGTTTAAAGC‐3′
RT‐qPCR			
Ribosomal protein S9	RPS9	5′‐CTGACGCTTGATGAGAAGGAC‐3′	5′‐CAGCTTCATCTTGCCCTCAT‐3′
Cadherin‐6	CDH6	5′‐AACACAGGCGACATACAGGC‐3′	5′‐TTGGACAACAAATGTACCGACA‐3′
SDF‐1	CXCL12	5′‐CCAAACTGTGCCCTTCAGAT‐3′	5′‐CGTCTTTGCCCTTTCATCTC‐3′
CD133	PROM1	5′‐GCAACAGCGATCAAGGAGAC‐3′	5′‐CACCAAGCACAGAGGGTCAT‐3′
VEGF	VEGFA	5′‐CATCACGAAGTGGTGAAGTTC‐3′	5′‐CACAGGATGGCTTGAAGATG‐3′

### Western blot analysis

Cells were rinsed twice with ice‐cold phosphate‐buffered saline (PBS; pH 7.0) with Ca^2+^ and Mg^2+^ and then collected on ice by scraping in cell lysis buffer (Cell Signaling Technology, Danvers, MA, USA) supplemented with protease inhibitor cocktail (Roche, Basel, Switzerland). Protein concentration was determined using the BCA Protein Assay (Pierce, MA, USA). The cell lysates were boiled at 95 °C for 5 min in SDS sample buffer (50 mm Tris–HCl, pH 6.8, 8% (v/v) Glycerol, 0.4% (v/v) sodium dodecyl sulfate (SDS), 0.005% (w/v) bromophenol blue, and 5% (v/v) β‐mercaptoethanol), and each cell lysate (20 μg) was resolved on SDS‐polyacrylamide gel electrophoresis (SDS/PAGE). For investigating cadherin‐6 knockdown in MSCs, siRNA‐transfected MSCs were rinsed twice with ice‐cold PBS and lysed with 2× SDS buffer (100 mm Tris–HCl, pH 6.8, 20% [v/v] glycerol, 2% [v/v] sodium dodecyl sulfate [SDS], 0.001% [w/v] bromophenol blue, and 10% [v/v] β‐mercaptoethanol) at 25 °C for 5 min. Cell lysates were collected by scrapping and denaturated at 95 °C for 5 min. Proteins were resolved on SDS/PAGE. Western blotting of proteins in the cell lysate was performed using standard procedures [19]. Primary antibodies against anticadherin‐6 (1 : 1000; R&D Systems, Minneapolis, MN, USA) and α‐tubulin (1 : 30 000–1 : 50 000, Sigma‐Aldrich, St. Louis, MO, USA) were used.

### Transwell migration assay

Serum‐starved MSCs were seeded onto transwell inserts (8 μm pore size, VWR International, Radnor, PA, USA) which were precoated with type 1‐P collagen (5 μg·mL^−1^, Nitta Gelatin NA Inc., Morrisville, NC, USA) using serum‐free media. After 6 h, the lower chamber media was replaced with serum‐free media containing SDF‐1 (50 ng·mL^−1^, R&D Systems) or conditioned media. When evaluating the migration of cadherin‐6‐knockdown MSCs, MSCs were transfected with either the control siRNA or cadherin‐6 siRNA for 12 h before serum starvation and then used for further analysis. To analyze the migration of MSCs overexpressing cadherin‐6, MSCs were transduced with a lentivirus encoding cadherin‐6.

### Lentiviral transduction

To prepare the caderin‐6 expressing vector (pLL‐CMV‐cadherin‐6‐GFP), the full‐length human cadherin‐6 was amplified from pCMV6‐AC‐GFP‐CDH6 (Origene) and cloned in pLL‐CMV‐GFP. The third generation of lentiviruses was produced in HEK293T cells by co‐transfecting pMDLg/pRRE, pRSV/REV, pBaEVRT, and transfer vector (pLL‐CMV‐cadherin‐6‐GFP or pLL‐CMV‐GFP).

### Three‐dimensional (3D) cell migration assay

Three‐dimensional migration was performed following the procedures described in the previous study [19]. A suspension containing MSCs transfected with cadherin‐6 siRNA or the control siRNA was prepared. This cell suspension was then mixed with type 1‐A collagen solution (1.5 mg·mL^−1^, Nitta Gelatin NA Inc.), and 3 μL of the collagen mixture was dispensed as droplets onto 12‐well plates. Following solidification, the MSCs were incubated in serum‐free media for 6 h. Subsequently, the media were replaced with the conditioned media, and the cells were allowed to migrate for 48 h. Migration analysis entailed the identification and quantification cells that migrated out from the collagen gel droplets. To investigate the effects of MSCs on U87 migration, U87 cells, either alone or co‐cultured for 3 days with MSCs on transwell inserts (0.4 μm pore size, Millipore, Burlington, MA, USA), were used for preparing cell suspension. After solidification of U87 cells and collagen gel, U87 cells were cultured with in serum‐free media for 6 h, and then, the media were replaced with fresh media including 0% or 10% FBS. U87 cells were allowed to migrate for 24 h.

### Colony formation assay

U87 cells were seeded on 6‐well plates at a density of 1 × 10^2^ cells per well. The cells were incubated for 14 days and subsequently fixed with 100% methanol for 10 min followed by staining with 0.5% crystal violet for 2 h. Colonies with a diameter exceeding 2 mm were then counted.

### Soft agar colony formation assay

U87 cells (2 × 10^3^) were mixed with 0.35% low‐gelling temperature agarose (Sigma‐Aldrich) in culture medium. This mixture was then seeded onto the top of 0.7% low‐gelling temperature agarose precoated 6‐well plate. The cells were cultured for 21 days, with the fresh culture media being added every 3 days.

### Sphere formation assay

U87 were seeded in a 24‐well low‐attachment plate (Corning Inc., Corning, NY, USA) using sphere‐forming assay media at a density of 2 × 10^3^ cells/0.8 mL. Fresh assay media were added to each well every 3 days and incubated until Day 7. The number of spheres with a diameter exceeding 50 μm was then counted.

### Vasculogenesis assay using a microfluidic cell culture chip

A microfluidic cell culture chip was fabricated with poly‐dimethyl‐siloxane (PDMS) [20]. For vasculogenesis assay, HUVECs, MSCs, and a single U87 sphere were combined with fibrinogen from bovine plasma (2.5 mg·mL^−1^, Sigma‐Aldrich). Thrombin (3.64 U·mL^−1^, Sigma‐Aldrich) was resuspended into the mixture just before the patterning. The mixture (7.5 μL) was added into the chip, and the chip was incubated at room temperature for 10 min for gelation. Subsequently, 200 μL of culture media was added into the media reservoir and incubated for 5 days. Then, the cells were fixed with 4% PFA and stained with DyLight 594 labeled Ulex Europaeus Agglutinin I (Vector, Newark, CA, USA).

### Statistical analysis

All data are presented as mean ± standard deviation (SD) and statistical significance was compared using an unpaired Student's *t*‐test. graphpad prism 9.4 (San Diego, CA, USA) was used for statistical tasks and *P*‐values < 0.05 were considered significant.

## Results

### Cadherin‐6 mediated the migration of MSCs in response to SDF‐1

**Fig. 1:**
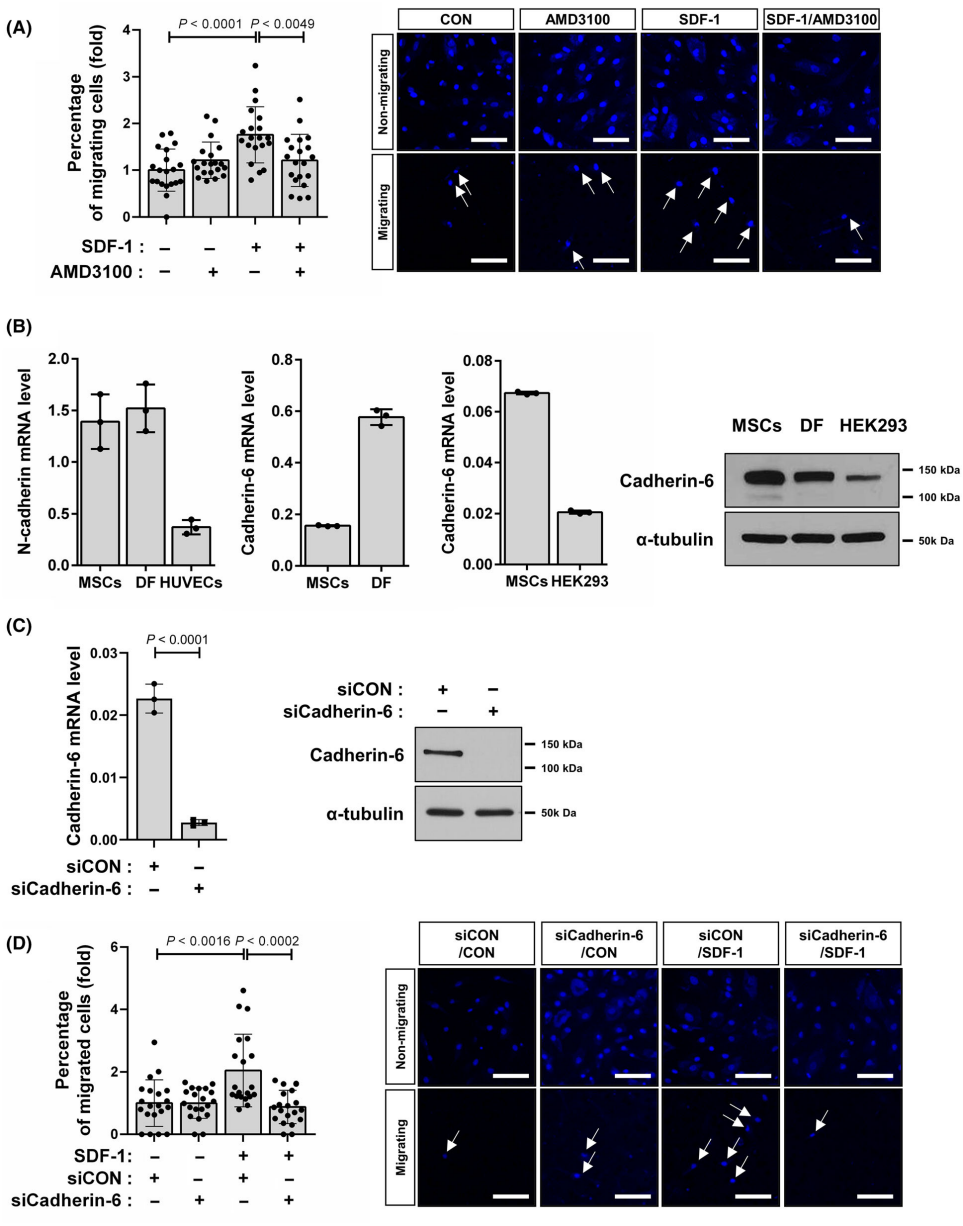
Cadherin‐6 mediated mesenchymal stem cell (MSC) migration in response to SDF‐1. (A) Transwell migration assay for quantifying MSC migration (*n* = 4 cultures from two independent experiments). MSCs were treated with SDF‐1 for 12 h. AMD3100 (10 μm) was pretreated for 30 min. The inserts were fixed with 4% paraformaldehyde and stained with DAPI. Cells on either the lower or upper surface of five randomly selected areas of each insert membrane were imaged using a confocal microscope. The number of cells in each image was counted, and the number of migrated cells was determined as a percentage of total cells on both sides of the insert. Representative images are shown. (B) RT‐qPCR to quantify mRNA expression level of N‐cadherin and cadherin‐6. (C) RT‐qPCR to quantify mRNA expression level of cadherin‐6 in MSCs transfected with cadherin‐6 siRNA (siCadherin‐6) or the control siRNA (siCON). (D) Transwell migration assay for quantifying the migration of MSCs transfected with cadherin‐6 siRNA (siCadherin‐6) or the control siRNA (siCON) (*n* = 4 cultures from four independent experiments). Representative images are shown. Data are shown as mean ± SD (*P*‐values by *t*‐tests). White arrows indicate the migrated MSCs on the lower surface of the membrane. Scale bar: 100 μm.

Mesenchymal stem cells migrated toward SDF‐1, and this migration was inhibited by AMD3100, an antagonist of CXCR4, which is a receptor for SDF‐1 (Fig. 1A, 1.8 ± 0.6 vs. 1.2 ± 0.6, *P* < 0.0049). We previously demonstrated that N‐cadherin mediates the collective migration of MSCs toward transforming growth factor beta (TGF‐β), breast tumor cells, and prostate tumor cells [13, 14, 21]. Cadherin‐6 exhibits its primary expression in the kidney and the central nervous system. During embryonic kidney development, cadherin‐6 shows high expression levels in mesenchymal cells [16]. Endothelial cells, including HUVECs, express N‐cadherin and fibroblasts [22, 23]. Dermal fibroblasts that are mesenchymal stromal cells expressed both N‐cadherin and cadherin‐6 (Fig. 1B). MSCs also expressed cadherin‐6 as well as N‐cadherin as dermal fibroblasts did (Fig. 1B). The expression level of cadherin‐6 mRNA in MSCs was comparable with that of HEK293 cells, kidney epithelial cells that have been demonstrated to express cadherin‐6 (Fig. 1B). Cadherin‐6 knockdown using siRNA (Fig. 1C) impaired the migration of MSCs in response to SDF‐1 (Fig. 1D, 2.0 ± 1.2 vs. 0.9 ± 0.5, *P* < 0.0002). Therefore, these results suggest that MSCs migrate in response to SDF‐1 in a SDF‐1‐dependent manner.

### Cadherin‐6 mediated the migration of MSCs toward glioblastoma cells

**Fig. 2:**
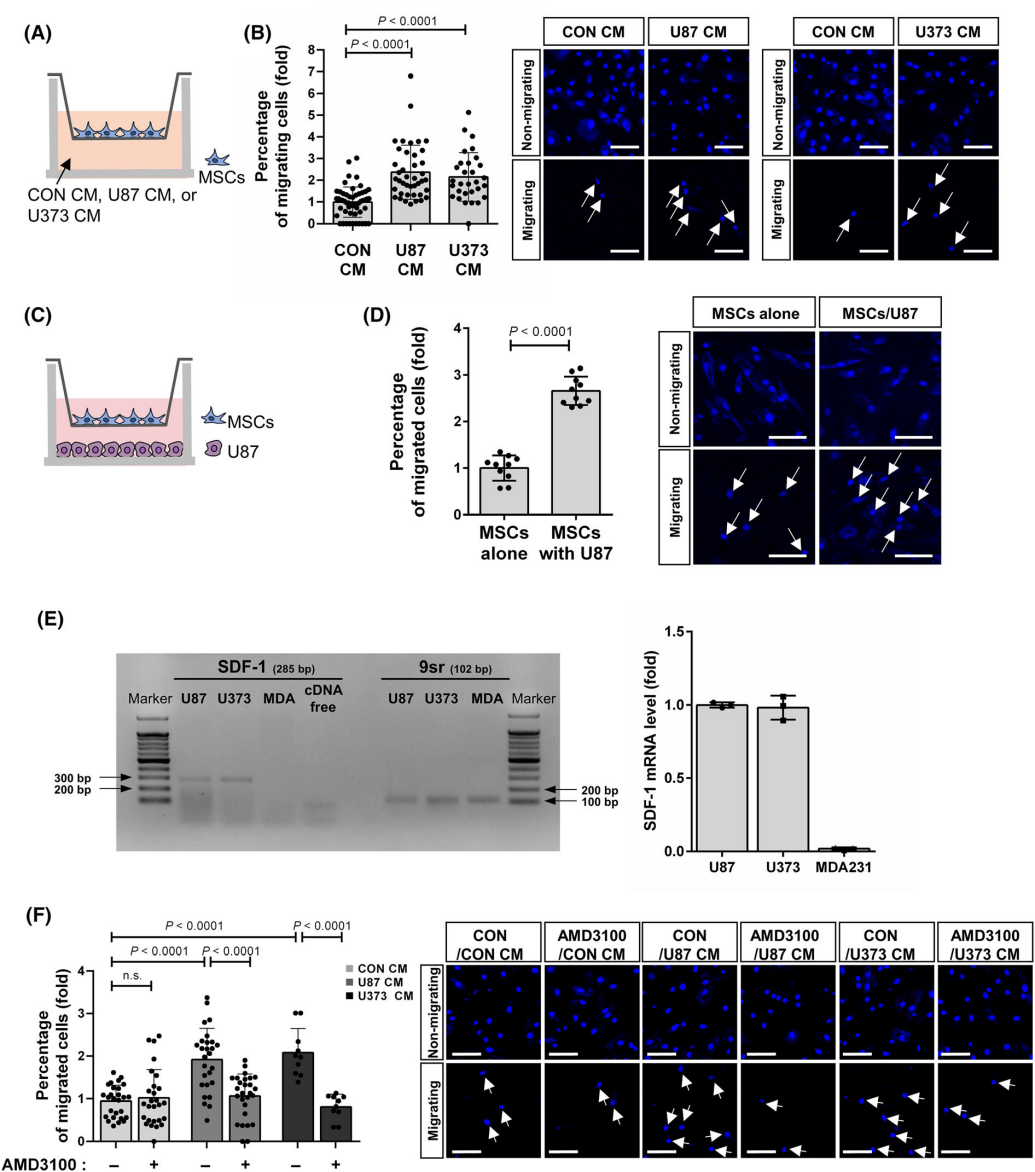
Mesenchymal stem cells (MSCs) migrated in response to glioblastoma cells dependently on SDF‐1. (A) Schematic representation of Transwell migration assay using the conditioned media. (B) Transwell migration assay for quantifying MSC migration in response to the conditioned media from U87 (U87 CM) and U373 (U373 CM) as well as the control conditioned media (CON CM) (*n* = 8 cultures from four independent experiments). MSCs were treated with the conditioned media for 12 h. (C) Schematic representation of Transwell migration assay conducted under a system of indirect co‐culture between MSCs and U87 cells. (D) Transwell migration assay for quantifying MSC migration in response to U87 cells (*n* = 2 cultures). MSCs were indirectly co‐cultured with U87 cells for 12 h using a membrane with 8 μm pore. (E) RT‐PCR and RT‐qPCR to evaluate expression levels of SDF‐1 in U87, U373, and MDA‐MB‐231 (MDA). (F) Transwell migration assay for quantifying SDF‐1 axis‐dependent migration of MSCs in response to U87 CM and U373 CM (*n* = 2 or 4 cultures). MSCs were treated with AMD3100 for 30 min prior to the incubation with the conditioned media. Representative images are shown. Data are shown as mean ± SD (*P‐*values by *t*‐tests). White arrows indicate the migrated MSCs on the lower surface of the membrane. Scale bar: 100 μm.

**Fig. 3:**
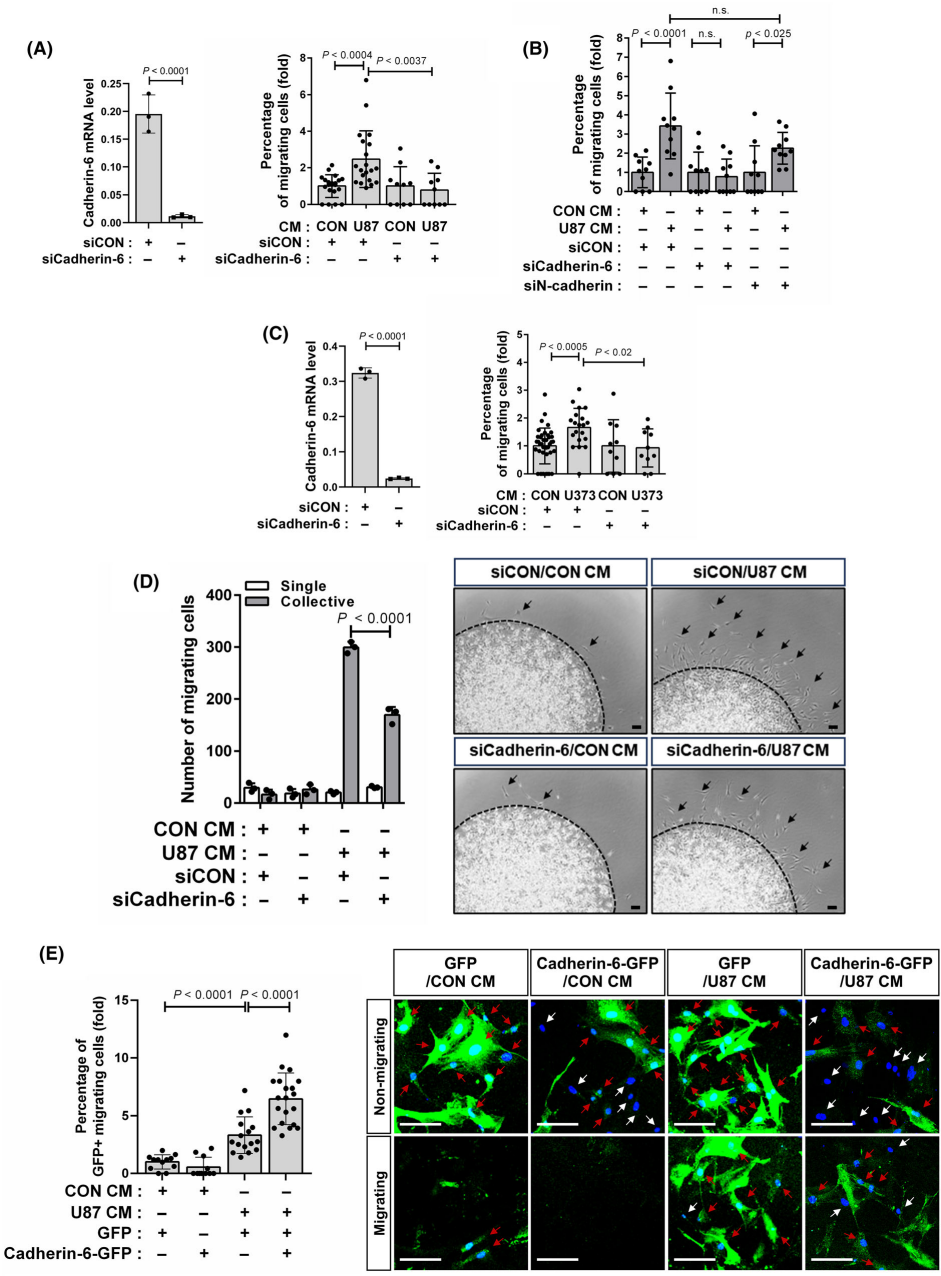
Cadherin‐6 mediated mesenchymal stem cell (MSC) migration in response to the glioblastoma cells. (A, B) Transwell migration assay for quantifying the migration of cadherin‐6 knockdown MSCs (A) or N‐cadherin knockdown MSCs (B) in response to U87 CM (*n* = 2 cultures for each). (C) Transwell migration assay for quantifying the migration of cadherin‐6 knockdown MSCs in response to U373 CM (*n* = 2 cultures). (D) 3D migration assay for quantifying the migration of cadherin‐6 knockdown MSCs in response to U87 CM. MSCs were treated with U87 CM for 12 h. Representative images are shown (*n* = 3 cultures). Black arrows indicate the migrating MSCs. Black dashed lines indicate the margin of collagen gel. (E) Transwell migration assay for quantifying MSCs expressing ectopic cadherin‐6 and GFP (Cadherin‐6‐GFP) or GFP in response to U87 CM (*n* = 4 cultures from two independent experiments). Representative images are shown. White arrows indicate MSCs without ectopic expression, while red arrows indicate MSCs with ectopic of GFP or of cadherin‐6 and GFP. Data are shown as mean ± SD (*P*‐values by *t*‐tests). Scale bar: 100 μm.

We investigated MSC migration toward conditioned media derived from glioblastoma cell lines, U87 and U373 (U87 CM and U373 CM, respectively). In comparison with control conditioned media, MSC migration was upregulated in response to U87 CM and U373 CM (Fig. 2A,B, CON CM vs. U87 CM: 1.0 ± 0.7 vs. 2.4 ± 1.3, *P* < 0.0001; CON CM vs. U373 CM: 1.0 ± 0.7 vs. 2.2 ± 1.1; *P* < 0.0001). Additionally, MSCs migrated toward U87 cells under the indirect co‐culture condition (Fig. 2C,D, 1.0 ± 0.3 vs. 2.7 ± 0.3, *P* < 0.0001). U87 cells and U373 cells expressed SDF‐1 (Fig. 2E). The expression was verified using a negative control, MDA‐MB‐231 breast cancer cell line that has been known not to express SDF‐1 [24]. Importantly, AMD3100, an antagonist of CXCR4, impaired MSC migration toward U87 CM (Fig. 2F, 1.9 ± 0.7 vs. 1.1 ± 0.5, *P* < 0.0001) and U373 CM (Fig. 2F, 2.1 ± 0.6 vs. 0.8 ± 0.3; *P* < 0.0001). These results suggest that MSCs migrated toward U87 and U373 via SDF‐1‐mediated mechanism. Then, we investigated whether cadherin‐6 mediates MSC migration toward glioblastoma cells. Cadherin‐6 knockdown MSCs exhibited decreased migration in response to U87 CM (Fig. 3A, 2.5 ± 1.6 vs. 0.8 ± 0.9, *P* < 0.0037; Fig. 3B, 3.4 ± 1.7 vs. 0.8 ± 0.9, *P* < 0.0004). However, N‐cadherin knockdown did not impair MSC migration toward U87 CM (Fig. 3B, 3.4 ± 1.7 vs. 2.3 ± 0.8, not significant). Additionally, cadherin‐6 knockdown MSCs also showed reduced migration in response to U373 CM (Fig. 3C, 1.7 ± 0.7 vs. 0.9 ± 0.7, *P* < 0.02). Cadherin‐6‐dependent migration of MSCs in response to glioblastoma cells was confirmed using a 3D cell migration assay [13, 14]. Cadherin‐6 knockdown led to a decrease in MSC migration from collagen gel in response to U87 CM (Fig. 3D, siCON+U87 CM collective vs. siCadherin‐6+U87 CM collective: 299.3 ± 11 vs. 169.7 ± 15.5, *P* < 0.0001). Importantly, most of the MSCs maintained cell–cell contacts during their migration toward U87 CM (Fig. 3D). The effect of cadherin‐6 overexpression on MSC migration was examined. In the experimental group, both cadherin‐6 and GFP were introduced into MSCs, whereas the control MSCs expressed GFP alone. The evaluation was based on the proportion of GFP‐positive MSCs that migrated to the lower membrane surface relative to the total number of GFP‐positive cells present on both the upper and lower membrane surfaces. Cadherin‐6 overexpression enhanced MSC migration toward U87 CM (Fig. 3E, GFP + U87 CM vs. Cadherin‐6‐GFP + U87 CM: 3.3 ± 1.6 vs. 6.5 ± 2.2, *P* < 0.0001), but it had no impact in response to the control conditioned media (Fig. 3E, GFP + CON CM vs. Cadherin‐6‐GFP + CON CM: 1.0 ± 0.6 vs. 0.5 ± 0.9, not significant). Consequently, these findings indicate that cadherin‐6 is necessary for MSC migration toward glioblastoma cells, although cadherin‐6 alone is not adequate for MSC migration.

### MSCs enhanced the vasculogenic capacity of glioblastoma cells

**Fig. 4:**
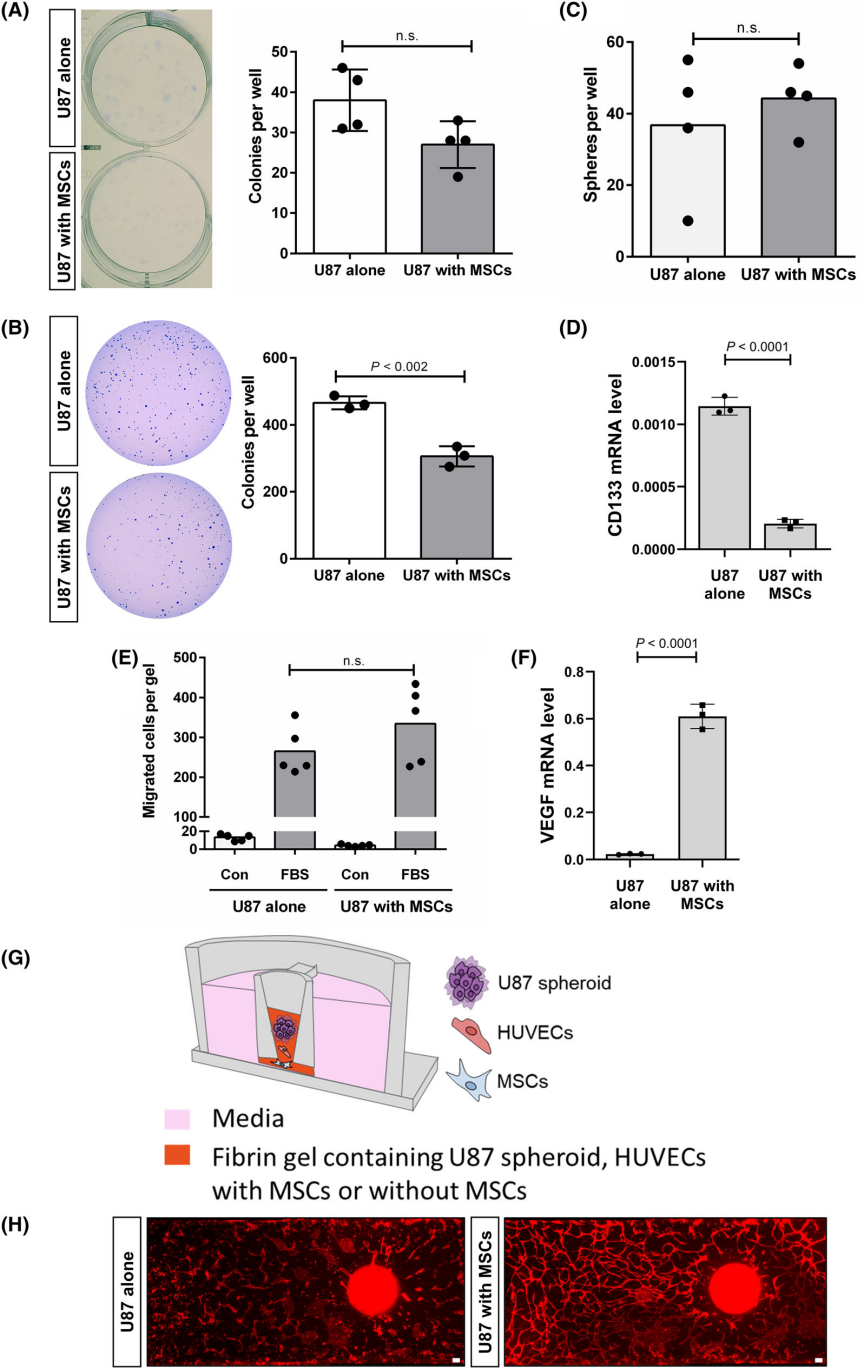
Mesenchymal stem cells (MSCs) enhanced the vasculogenic capacity of U87 cells. (A) Colony formation assay (A), soft agar colony formation assay (B), and sphere formation assay (C) of U87 cells after indirect co‐culture with MSCs (U87 with MSCs), compared with the control U87 cells (U87 alone). (D) RT‐qPCR to quantify mRNA expression level of CD133 in U87 after indirect co‐culture with MSCs. (E) 3D cell migration assay for quantifying the migration of U87 cells in response to 10% FBS after indirect co‐culture with MSCs. (F) RT‐qPCR to quantify mRNA expression level of VEGF in U87 after indirect co‐culture with MSCs. (G) Photograph of the microfluidic cell culture chip and a schematic drawing of experimental procedure. (H) Vascular network formation in fibrin gels with HUVECs, U87 spheroid, and either MSCs (U87 with MSCs) or in fibrin gels with HUVECs and U87 spheroids (U87 alone). Data are shown as mean ± SD (*P*‐values by *t*‐tests). Scale bar: 100 μm.

Mesenchymal stem cells have been shown to contribute to the maintenance of glioblastoma stem cells and enhance both the proliferation and migration of glioblastoma cells [2, 25, 26]. To investigate the effects of MSCs on glioblastoma cells, the characteristics of U87 cells which had been indirectly co‐cultured with MSCs were analyzed. MSCs did not increase the cellular proliferation of U87 cells or their anchorage‐independent proliferation. MSCs did not alter the colony‐forming ability of U87 cells (Fig. 4A, U87 alone: 38.0 ± 7.6, U87 with MSCs: 27.0 ± 5.8). MSCs impaired the ability to form colonies in soft agar (Fig. 4B, U87 alone: 466.3 ± 19.6, U87 with MSCs: 306.0 ± 30.0; *P* < 0.002). In addition, the introduction of MSCs did not lead to an increase in traits typically associated with the cancer stem cell phenotype in U87 cells. There was no difference in the ability of sphere formation between U87 cells co‐cultured with MSCs and the control U87 (Fig. 4C, U87 alone: 39.3 ± 9.9, U87 with MSCs: 44.3 ± 9.1). The expression of CD133, a marker associated with cancer stem cell characteristics, decreased in U87 cells co‐cultured with MSCs compared with the control (Fig. 4D, U87 alone: 0.0011 ± 0.00007, U87 with MSCs: 0.0008 ± 0.00003; *P* < 0.0001). The migration capacity of U87 did not increase after indirect co‐culture with MSCs. U87 cells co‐cultured with MSCs exhibited a similar migration capacity in response to FBS compared with control U87 cells (Fig. 4E, Con and U87 alone: 13.2 ± 3.5, FBS and U87 alone: 265.2 ± 60.1, Con and U87 with MSCs: 4.4 ± 1.1, FBS and U87 with MSCs: 334.4 ± 95.7). Therefore, the results suggest that MSCs lack the ability to maintain the cancer stem cell phenotype of U87 cells and increase the proliferation and migration of U87. Tumor vascularity is one of the significant characteristics of glioblastoma. Conflicting effects of MSCs on tumor vascularity have been reported; MSCs can impair angiogenesis in glioblastoma [27], while also enhancing neovascularization [6]. VEGF has been known to play a crucial role in tumor vascularity in glioblastoma [28]. A high level of VEGF has been associated with poor overall survival in glioblastoma patients [29]. U87 cells indirectly co‐cultured with MSCs exhibited higher VEGF expression level compared with the control U87 cells (Fig. 4F, U87 alone: 0.02 ± 0.002, U87 with MSCs: 0.61 ± 0.052; *P* < 0.0001). Subsequently, a vasculogenesis assay was conducted using a microfluidic cell culture chip [20] (Fig. 4G). Under the condition where MSCs co‐existed, U87 cells enhanced the formation of blood vessel‐like tube structures in HUVECs, compared with U87 cells alone (Fig. 4H). Therefore, these results suggest that MSCs upregulate the vasculogenic capacity of U87 cells.

## Discussion

In this study, we found that cadherin‐6 plays a crucial role in mediating MSC migration toward both SDF‐1 and glioblastoma cells. Cadherin‐6 knockdown reduced MSC migration toward SDF‐1 and conditioned media from glioblastoma cell lines, which mimic the glioblastoma microenvironment. Additionally, MSCs were observed to enhance the vasculogenic capacity of glioblastoma cells. The current study expands understanding of the intricate interplay between glioblastoma and MSCs. By promoting vascularization, MSCs exerted an influence on the pathological features of glioblastoma, opening up new possibilities for therapeutic strategies that aim to target glioblastoma eradication. Furthermore, these results suggest that the current strategy of utilizing MSCs as carriers for antiglioblastoma drugs requires careful examination.

The interaction between mesenchymal stem cells and SDF‐1 plays a pivotal role in the tumor microenvironment, exerting significant influence on tumor progression across various carcinoma types. SDF‐1, expressed from tumor cells, has been shown to facilitate the recruitment of mesenchymal stem cells to the tumor microenvironment [30, 31]. Once recruited, mesenchymal stem cells secrete SDF‐1, which in turn enhances the epithelial‐mesenchymal transition (EMT) of prostate cancer cells [32]. In breast cancer, tumor cells have been found to induce the expression of SDF‐1 in mesenchymal stem cells. This induction consequently promotes tumor cell proliferation through the upregulation of SDF‐1 within the tumor microenvironment [33, 34]. Additionally, SDF‐1 serves as a mediator for the migration of mesenchymal stem cells toward glioblastoma cells [35].

Mesenchymal stem cells expressed both N‐cadherin and cadherin‐6. MSCs have been shown to migrate toward TGF‐β, breast cancer cells, and prostate cancer cells in a manner dependent on N‐cadherin [13, 14, 21]. For the development of techniques aimed at controlling the target‐specific migration of MSCs, it is crucial to ascertain whether different cadherins are selectively and specifically required for MSCs to migrate in response to various migratory stimuli. However, it remains unclear whether cadherin‐6 can mediate MSC migration toward TGF‐β and breast tumor cells or if N‐cadherin mediates their migration toward SDF‐1 as well as toward glioblastoma cells. Further investigation is needed to clarify these aspects. N‐cadherin was found to mediate the collective migration of MSCs in response to breast tumor cells and prostate tumor cells [13, 14]. The number of MSCs that maintained their cell–cell contacts during their migration in response to the conditioned media from U87 glioblastoma cells increased, compared with the control conditioned media. However, there was no change in the number of MSCs that migrated without the cell–cell contacts. Additionally, cadherin‐6 knockdown downregulated the number of MSCs that collectively migrated in response to U87 by maintaining their cell–cell contacts, but did not affect the number of MSCs migrating as single cells (Fig. 3D). The results highlight the potential role of cadherin‐6 in regulating cell–cell contacts during MSC migration, opening avenues for further investigation in this area. Understanding the distinct contributions of N‐cadherin and cadherin‐6 in mediating MSC migration in response to different cues may be crucial for developing targeted antitumor techniques supported by MSC‐based therapies.

Mesenchymal stem cells have been known to exhibit contradictory effects on glioblastoma. Mesenchymal stem cells have been characterized alternatively as both promoters and inhibitors of tumor growth, with conflicting findings stemming from variations in types and sources of mesenchymal stem cells, the utilization of different glioblastoma cell lines or glioblastoma stem cells, and the choice between *in vitro* and *in vivo* models. The ambiguity surrounding the precise role of mesenchymal stem cells in glioblastoma progression has been exacerbated by the diverse outcomes observed, whether through direct interactions via co‐cultures or indirect mechanisms involving mesenchymal stem cells‐secreted factors or extracellular vesicles [2, 26]. Mesenchymal stem cells have been shown to enhance the proliferation of glioblastoma cell lines [36] or reduce it via cell cycle arrest, dependent on the decrease in cyclin D [37]. They have also been observed to both enhance and downregulate the migration of glioblastoma cell lines [36, 38, 39]. Additionally, mesenchymal stem cells have a regulatory role in the tumor vascularity of glioblastoma. Conflicting roles have been observed in this context, as mesenchymal stem cells have been shown to both inhibit angiogenesis by downregulating the expression of angiogenic factors in glioblastoma cells and promote neovascularization by fusing with glioblastoma stem cells [6, 26, 27]. The current study showed that mesenchymal stem cells enhanced the angiogenic characteristics of U87 glioblastoma cells by increasing VEGF, one of the most crucial pro‐angiogenic factors, without affecting cellular proliferation, migration, or the characteristics of glioblastoma cancer stem cells. Additionally, using the microfluidic cell culture chip, this study demonstrated that mesenchymal stem cells increased the vasculogenic capacity of U87 cells. However, it is still unclear whether mesenchymal stem cells can regulate the expression of antiangiogenic factors in glioblastoma cells or if mesenchymal stem cells can modulate their own pro‐angiogenic and antiangiogenic factors in response to glioblastoma microenvironment. It also needs to be investigated whether mesenchymal stem cells can directly affect HUVECs regardless of any potential effects of U87 cells on HUVECs. Further research is necessary to address these questions. It is important to note that the behavior of mesenchymal stem cells within tumor microenvironments is highly complex and context‐dependent. Factors such as the heterogeneity of tumor cells, variations in mesenchymal stem cell properties, and the dynamic nature of cell–cell interactions within the microenvironment may contribute to the observed outcomes. Given the complexity of these interactions, further investigations are warranted to elucidate why mesenchymal stem cells did not enhance proliferation, migration, or cancer stem cell phenotypes (Fig. 4).

## Conflict of interest

The authors declare that the research was conducted in the absence of any commercial or financial relationships that could be construed as a potential conflict of interest.

## Author contributions

AP, S‐EK, and K‐SP designed the experiments; AP, S‐EK, JY, and DS performed the experiments. AP and K‐SP analyzed the data, and wrote and revised the manuscript. K‐SK and EK reviewed the manuscript. All authors contributed to the article and approved the final version submitted for publication.

## Data Availability

All data generated or analyzed during this study are included in this article.
